# A New Way Forward: How Brain SPECT Imaging Can Improve Outcomes and Transform Mental Health Care Into Brain Health Care

**DOI:** 10.3389/fpsyt.2021.715315

**Published:** 2021-12-10

**Authors:** Daniel G. Amen, Michael Easton

**Affiliations:** ^1^Amen Clinics, Costa Mesa, CA, United States; ^2^Department of Psychiatry, Rush University Medical Center, Chicago, IL, United States

**Keywords:** brain SPECT, evidence-based, brain trauma, dementia, hypofrontality, hyperfrontality, complex cases, brain health

## Abstract

In the past three decades, brain single-photon-emission-computed-tomography (SPECT) imaging has garnered a significant, evidence-based foundation for a wide array of indications relevant to the field of clinical psychiatry, including dementia, traumatic brain injuries, seizures, cerebrovascular disease, complex neuropsychiatric presentations, and treatment-resistant disorders. In clinical psychiatric practice, however, SPECT remains underutilized. Only a small percentage of psychiatric clinicians use brain imaging technology. In this article, the authors provide a rationale for shifting the paradigm to one that includes broader use of SPECT in the clinical psychiatric setting, primarily for patients with complex conditions. This paper will outline seven specific clinical applications. Adding neuroimaging tools like SPECT to day-to-day clinical practice can help move psychiatry forward by transforming mental health care, which can be stigmatizing and often shunned by the general public, to brain health care, which the authors argue will be more likely to be embraced by a larger group of people in need.

## Introduction

Despite the early enthusiasm in the late ‘80s and early ‘90s about the use of functional neuroimaging in psychiatric practice, if you were to ask the majority of psychiatrists today why they don't utilize it, their response would most likely be, “there are no adequate large-scale studies demonstrating the validity of this in helping with psychiatric diagnosis and treatment.” Yet there are nearly 15,000 references on www.pubmed.gov about brain single-photon-emission-computed-tomography (SPECT) imaging encompassing a wide variety of neuropsychiatric indications (1). Is there another reason clinicians are not using brain SPECT?

In 1992, Holman and Devous authored an important article titled “Functional Brain SPECT: The Emergence of a Powerful Clinical Method,” which articulated the promise of functional brain imaging: “*SPECT techniques provide a powerful window into the function of the brain and promise to become an important component of the routine clinical evaluation of patients with neurological and psychiatric diseases* (2).”

In 1992 and 1993, the American Psychiatric Association's (APA) Annual Meeting on SPECT imaging in psychiatry included day-long courses, symposia, and workshops covering the technology. After reading Holman and Devous' seminal paper and attending those APA meetings, author DA began utilizing brain SPECT imaging in clinical practice for a range of neuropsychiatric indications. Subsequently, he has amassed a database of more than 194,000 brain SPECT scans on patients with a vast array of complex neuropsychiatric conditions. Since the publication of Holman and Devous' paper in 1992, a wealth of scientific research has been published that lends support to the use of brain SPECT in psychiatric clinical practice. For example, in a 1996 issue of the Harvard Review of Psychiatry, Vasile wrote, “*The clinical utility of SPECT in neuropsychiatry is well established, and research devoted to its use in primary psychiatric disorders has been gaining momentum* (3).” In 2001, Camargo asserted that, “*Brain SPECT … is rapidly becoming a clinical tool in many places. The importance of this technique in nuclear medicine today should not be overlooked, particularly in cerebrovascular diseases, dementias, epilepsy, head injury, malignant brain tumors, movement disorders, obsessive-compulsive disorder, Gilles de la Tourette's syndrome, schizophrenia, depression, panic disorder, and drug abuse* (4).”

Although this evidence-based tool that is relevant to diagnosing and treating a multitude of psychiatric conditions has been available for three decades, only a small segment of psychiatrists worldwide have adopted the use of SPECT in clinical practice. Thus, the field of psychiatry continues to be the sole medical specialty that typically does not look at the organ it treats. In no other field of medicine do physicians make diagnoses or treatment recommendations without biological information. Psychiatry's refusal to widely adopt SPECT and/or other functional brain imaging tools—such as quantitative electroencephalography (qEEG), positron emission tomography (PET), or arterial spin labeling (ASL)—not only compromises its credibility as a branch of medical practice, but it also harms patients who suffer due to diagnoses or treatments that are inaccurate, inadequate, and/or stigmatizing and discouraging.

## Why Psychiatry Failed to Adopt SPECT

One of the primary reasons why psychiatry has failed to adopt SPECT in clinical practice over the past three decades lies in the medical specialty's adherence to categorical diagnostic nomenclature in the Diagnostic and Statistical Manual of Mental Disorders (DSM). When SPECT began generating excitement in the field of psychiatry in the late 1980s and early 1990s, researchers made attempts to match brain patterns seen on SPECT with specific diagnoses found in the DSM. When the scientific investigators failed to pinpoint consistent DSM patterns for mental health conditions, they discarded neuroimaging for clinical practice, claiming it was not yet a useful tool (5, 6). In hindsight, the problem likely did not lie in the neuroimaging tools, including SPECT, PET, and qEEG. These sophisticated brain imaging technologies accurately assess brain function in patients. It is more likely that the problem arose from psychiatry's diagnostic methodology, which presumed that the patterns seen in neuroimaging would correlate directly to the diagnostic categories found in the DSM.

Thomas Insel, the former Director of the National Institutes of Mental Health, said in his 2005 keynote speech to the American Psychiatric Association, “*The DSM-IV has 100% reliability and 0% validity…We need to develop biomarkers, including brain imaging, to develop the validity of these disorders…Trial-and-error diagnosis will move to an era where we understand the underlying biology of mental disorders … We are going to have to use neuroimaging to begin to identify the systems pathology … to develop treatments that go after the core pathology, understood by imaging* (7).” Unfortunately, psychiatrists have not yet moved beyond looking only at symptom clusters which have failed to demonstrate clear separation of distinct illnesses but have high levels of comorbidity in epidemiological, clinical, and genetic studies (8, 9).

In 2005, Dr. Insel also noted in writing, “…*patterns of regional brain activity associated with normal and pathological mental experience can be visualized … and ultimately, biomarkers for mental disorders may not be proteins or neurotransmitters but may emerge from neuroimaging (functional magnetic resonance imaging* (fMRI), *single photon emission computed tomography* (SPECT)*, etc.). Logically, if these are disorders of brain systems, then the visualization of abnormal patterns of brain activity should detect the pathology of these illnesses* (10).”

A psychiatrist trying to identify a singular neuroimaging pattern for major depressive disorder is similar to a cardiologist searching for a sole imaging pattern for angina. Just as there are a wide variety of causes and imaging patterns seen in patients with angina, there are also a multitude of causes and neuroimaging patterns seen in psychiatric conditions, such as major depressive disorder, attention-deficit hyperactivity disorder (ADHD), bipolar disorder, schizophrenia, and autism spectrum disorder (ASD). We can see five individuals with the same DSM diagnosis yet see five very different SPECT scans. Why would we treat them all the same? These individuals should not be expected to respond to similar interventions. We would miss important information by looking only at symptoms. As an adjunct tool, SPECT can inform the direction of treatment more precisely than a one-size-fits-all DSM-V diagnosis can. It has been shown that by utilizing functional brain imaging we can improve treatment decisions and outcomes (11, 12). Yet, the use of neuroimaging is still disregarded by traditional medical specialties.

Consider these two case examples of how mainstream psychiatry fails in an effort to treat complex patients without the use of SPECT neuroimaging:

Mr. C suffered from crippling anxiety, extreme mood swings, recurring panic attacks, negative thinking patterns, anger issues, and difficulty sleeping. These issues negatively impacted his relationships, both at work and in his social life. He was shy and reported always being in a bad mood. In his family, there was a history of addictions and depression. Mr. C first saw a psychiatrist when he was a teenager and was diagnosed with multiple disorders, including bipolar disorder, ADHD, and intermittent explosive disorder (IED).

**Figure 1:**
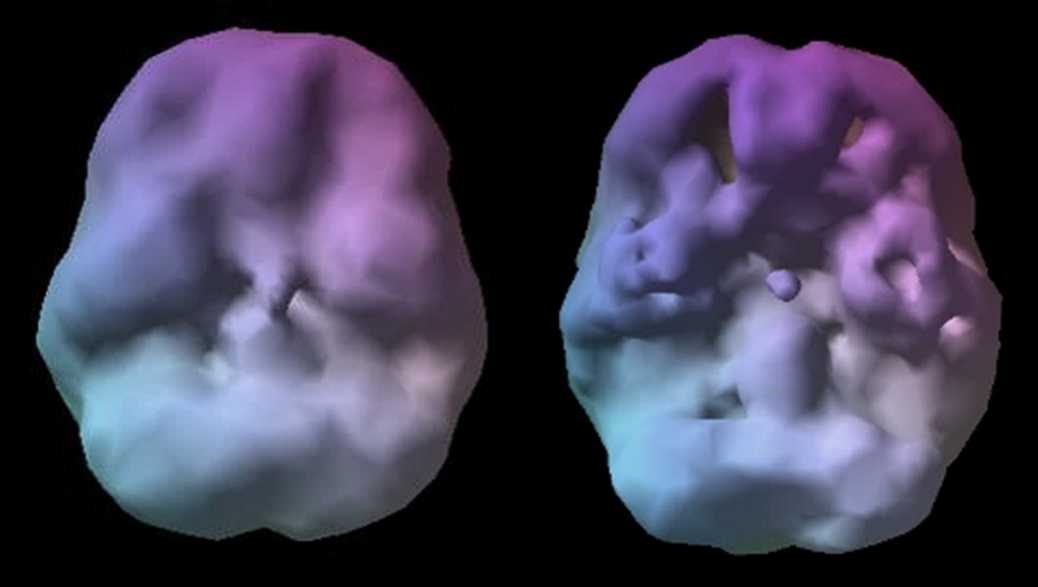
Healthy SPECT image compared to C's pre-treatment SPECT image. A healthy SPECT image (L) shows full, even, symmetrical activity compared to C's pre-treatment SPECT image (R) with low activity, especially in the prefrontal cortex and temporal lobes. Photon emission was captured using a high-resolution Picker (Phillips) Prism 3,000 triple-headed gamma camera with fan beam collimator with data collected in 128 × 128 matrices, yielding 120 images per scan with each image separated by three degrees spanning 360 degrees. A low pass filter was applied with a high cutoff. A Chang attenuation correction was performed using linear methods. All images were processed using Odyssey software (Picker), with transaxial slices oriented horizontal to the AC-PC line. Coronal, sagittal, and transaxial slice images (6.6 mm apart, unsmoothed) were then rendered in the Odyssey step-20 scale. Image acquisition and processing methods apply to all images presented in this article.

**Figure 2:**
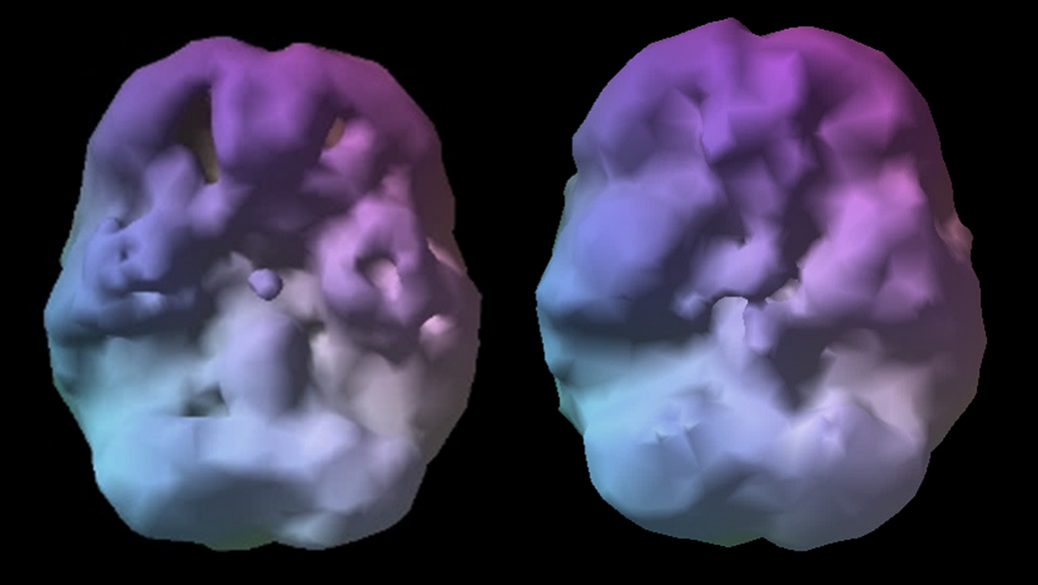
C's SPECT surface underside view images before (L) and after (R) treatment. C's SPECT image before treatment (L) reveals low activity, especially in the prefrontal cortex and temporal lobes compared to post-treatment image (R) showing overall improvement.

In the years following his diagnoses, Mr. C tried a variety of medications in an attempt to stabilize his moods. The various prescriptions came with a host of detrimental side effects that exacerbated things and led to an 80-pound weight gain. When coupled with his social anxiety, the added weight pushed him further into isolation. He became unable to work and his family referred him to author DA for a comprehensive evaluation, including SPECT imaging. His SPECT scan (see Figures 1, 2) showed significantly decreased overall cerebral blood flow, most pronounced in the prefrontal cortex (PFC) and temporal lobes. The blood flow and activity patterns seen on his scan were indicative of a past head injury and exposure to toxins. These findings led to additional questions to find the root causes for the abnormal blood flow and activity in his brain.

Mr. C eventually divulged that he grew up in a family that owned and operated a motor speedway. Since childhood, he had been racing cars and has spent substantial amounts of time around the speedway where he was exposed to toxic fumes from gasoline. In addition, he had experienced multiple concussions, one of which resulted from crashing into a wall while racing a car.

After reviewing his scans and learning more about his history, it was clear Mr. C was struggling with the lingering impacts from multiple head injuries as well as the exposure to toxic gasoline fumes and had previously been given psychiatric labels based on symptom clusters. His psychiatric medications were stopped, and he was given brain supportive supplements and placed on a program to rehabilitate his brain with diet, exercise, and hyperbaric oxygen therapy shown to help with traumatic brain injury (13). Mr. C came to see his problem as a brain health issue, rather than as a mental health issue, which shifted his mindset to being a more active participant in his own care. In just a few months, he lost 80 pounds, his mood was better, anxiety was decreased, and he had better control over his temper. Several months later, his brain also showed significant improvement on the follow-up SPECT scan. After seeing his brain SPECT scan and learning about why his brain looked so troubled, Mr. C made the decision to give up car racing.

This case demonstrates how functional neuroimaging can be a powerful tool helping to show that other factors are aggravating or are the primary cause of neuropsychiatric syndromes. As seen above, traumatic brain injury as well as toxic exposure are examples of causative factors that most psychiatrists would not generally look for. The most commonly unidentified contributor to psychiatric disorders is traumatic brain injury (TBI). Although there is substantial evidence indicating its role in neuropsychiatric conditions, TBI frequently goes unrecognized and untreated (14–17). SPECT gives us clues to help identify problems that were previously unseen and undiagnosed.

A common problem seen in clinical practice is that of patients who, after years of remission, become non-responders later in their lives. Functional imaging can help identify factors contributing to this.

**Figure 3:**
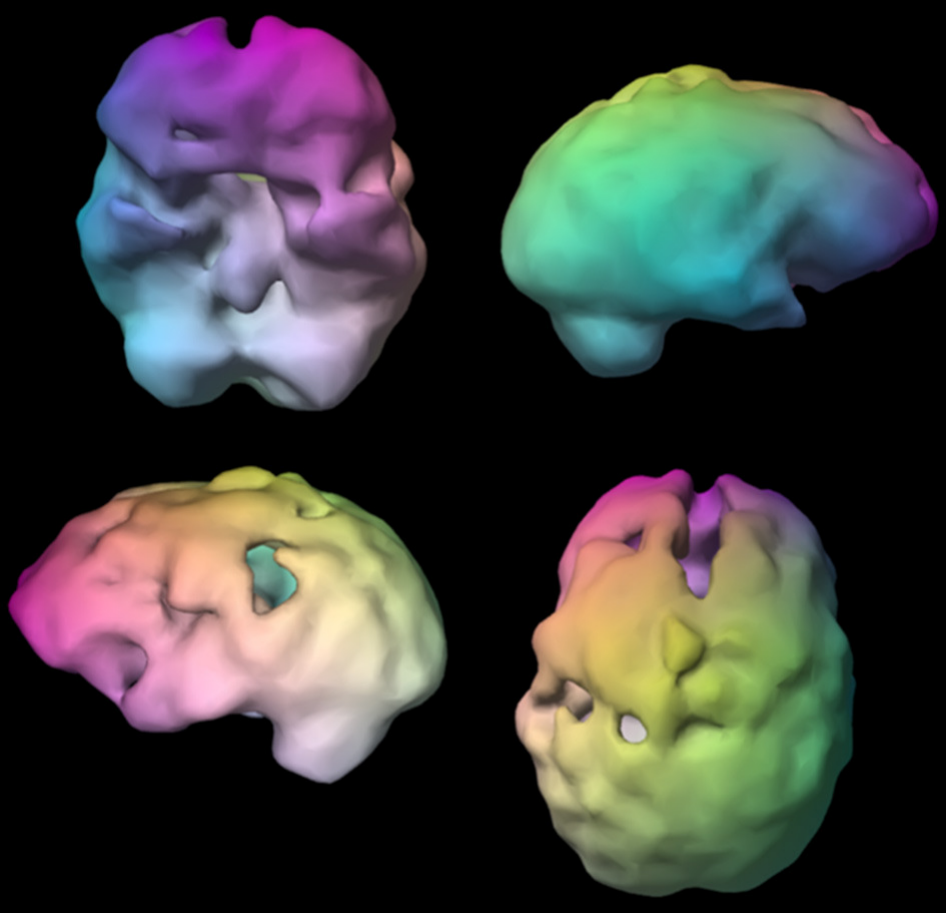
Mr. O's surface view images. Note decrease perfusion in temporal lobes, longitudinal fissure, and parietal regions, as well as findings suggestive of ventricular dilation.

Mr. O was a 77-year-old gentleman whose bipolar depression had been treated successfully for years but more recently had become progressively more difficult to treat. He no longer responded to various medications and had converted from being an excellent ECT responder to a non-responder. Upon looking at his SPECT scan, he presented with significant decreased perfusion in his temporal lobes, longitudinal fissure, and parietal regions (see Figure 3). It became clear that there was a potential history of brain injury as well as a possible neurodegenerative process contributing to his poor response. Instead of treating him with higher doses or more medications, he was continued on his current medication regiment, and was placed on brain supportive supplements and given a program to improve his brain health with diet and exercise, as well as a recommendation for hyperbaric oxygen therapy. With this treatment strategy, Mr. O converted back to being a treatment responder along with the family recognizing the need to become more involved in his care.

As with thousands of other patients who found relief of their symptoms because of the additional data provided by SPECT neuroimaging, had that not been incorporated, the underlying perfusion abnormalities in these patients would have gone undetected, thus denying them the opportunity to reduce their symptoms.

## The Challenges of Shifting a Paradigm

There is expanding knowledge about the influence various brain regions have on neuropsychiatric function and how changes in these areas can result in predictable symptoms. Yet little of this information has crossed over into clinical practice. Part of the problem is the neurobiology of psychiatric disorders do not typically line up with the Diagnostic Statistical Manuel (DSM), which defines illnesses as symptom clusters that should be distinguishable conditions with common underlying etiologies. In actuality, each DSM diagnosis represents a heterogenous group of illnesses with a variety of underly etiologies. Given the DSM has been the foundation of subject choice in most clinical research it is unlikely it will correlate with functional imaging. The authors believe this is the main reason neuroimaging has not been incorporated into clinical psychiatric practice (18).

Think about the many case conferences we've all attended. A group of psychiatrists looking at symptom clusters without looking at brain function, all of whom see the same patient and use their subjective impressions of an individual's symptoms (all too frequently influenced by psychodynamic interpretations) to come up with a variety of different diagnoses and treatment recommendations. To complicate matters further, these recommendations are all based on research evaluating a heterogeneous group as if they are a single illness. Clinical course and treatment response for a particular DSM diagnosis cannot be predictive using this methodology. It is not a surprise that the overall response and remission rates demonstrated in pharmacotherapeutic studies are low when <40% of the DSM-V diagnoses meet validity testing standards (19–21).

If psychiatry is to move forward, we must first challenge the use of the categorical DSM system as the only way to diagnose patients and learn how to incorporate the findings of evidence-based neuroimaging.

As is the case with any evolving clinical science, there is a need for more work to expand our knowledge of SPECT's benefits and limitations within the clinical setting. In this article, the authors detail the evidence-based rationale for more widespread use of SPECT in psychiatric clinical practice. It explores a number of the reasons why SPECT was derailed despite showing so much promise since the late 1980s and early 1990s, reviews the obstacles and limitations involved with utilizing SPECT, and presents numerous indications that clinical psychiatrists can use immediately. This article primarily looks at SPECT as opposed to other functional neuroimaging options for the following five reasons. First, the authors have extensive experience with SPECT, and as previously stated, have built a growing database of brain scans that currently totals over 194,000 scans. Second, all major hospitals throughout North America, South America, Asia, and Europe are equipped with SPECT cameras, which means it is already a neuroimaging tool that is widely available. Third, a growing body of scientific literature validates the usefulness of brain SPECT imaging for a variety of issues within the field of clinical psychiatry. Fourth, several expert review bodies have endorsed SPECT for numerous indications that are relevant to clinicians, including dementia and brain trauma. Fifth, compared with other neuroimaging tools, SPECT is typically a less expensive imaging modality and has been recognized by the health insurance industry in the United States with specific reimbursement codes for over three decades, although reimbursement is unpredictable, especially for purely psychiatric indications.

In 1962, scientific historian and philosopher, Thomas Kuhn, wrote that scientific revolutions typically occur in 5 stages (22).

### Stage I: The Discrepancies Show

*In the first stage, the revolution is started when the standard paradigm begins to fail*. For example, when author DA would use DSM criteria to diagnose patients with major depression or ADHD and put them on standard treatments, such as fluoxetine or methylphenidate, some patients became suicidal or aggressive. This was a paradigm-based failure that occurred far too often and was traumatic for patients and caregivers.

### Stage II: The Disagreements Start

*Once the paradigm begins to fail, experts begin to look for ways to fix their theories, but they resist discarding their old models entirely and instead look for small fixes*. Over time, the failing model splinters into many competing schools of thought. Kuhn wrote that no matter how wrong their models have become, the leaders maintain their beliefs and continue trying to tweak their ideas to preserve their power and influence. There are now six versions of the DSM, which has not been substantially overhauled since 1980's DSM-III.

### Stage III: The Revolution

*Over time, a new paradigm emerges that resolves many of the problems in the field*. A new paradigm can be: “Most psychiatric illnesses are not mental health issues at all. Neuroimaging clearly shows they are brain health issues that steal the mind (23).” It reinterprets existing knowledge while retaining the best of the old thinking and integrating the latest knowledge into a fresh model, thereby creating a paradigm shift.

### Stage IV: The Rejection

*The new paradigm is then rejected and ridiculed by the leaders in the field*. This is one of the most reliable stages of a scientific revolution. The old guard becomes frustrated that the new idea did not come from them, and because they hold tightly to their own theories, this period may last for decades until they retire or die. Max Planck, the noted Nobel Laureate in physics, once wrote, “*A new scientific truth does not triumph by convincing its opponents and making them see the light, but rather because its opponents eventually die, and a new generation grows up that is familiar with it*^1^*.”* Science advances through funerals.

### Stage V: The Acceptance

*The new theory is adopted gradually as younger, more open-minded scientists accept it early in their careers and later become the leaders of the field*. Kuhn also noted that new paradigms are often championed by professionals who are outsiders and not wed to the status quo.

## Evidence-Based Medicine: Brain Spect Imaging

Structural neuroimaging, such as MRI or CT scans, is routinely recommended for first-break psychoses. “*The use of anatomical scans of individuals with recent onset psychosis is justified in order to rule out neurological diagnoses that may mimic schizophrenia in their early stages. A scan also serves to reassure patient, family, and physician that diagnostic possibilities with visible cerebral insult have been considered* (24)*.”* Despite this usage, research shows that such anatomical brain scans impact clinical decisions in only approximately one-half of 1% of all cases, which shows their clinical value is sorely limited (25). As evidenced in a 2-year review, when ordered appropriately in a hospital setting, SPECT provides significantly higher levels of information that is clinically relevant in complex mental health conditions (26).

Currently, the scientific literature on functional brain imaging related to conditions that are relevant to clinical psychiatry numbers in the tens of thousands and involves hundreds of thousands of patients^2^. The abundance of peer-reviewed research involving SPECT validates its reliability as a measure of brain function, and in particular, regional cerebral blood flow (rCBF). Well-respected medical organizations, including the American College of Radiology (ACR) (27), the Canadian Association of Nuclear Medicine (28), and the European Society of Nuclear Medicine (ESNM) (29), have reported evidence-based medicine (EBM) clinical indications for the use of brain SPECT imaging in the evaluation of patients. Generally accepted clinical indications for SPECT include, but are not limited to:

- Evaluation of patients with suspected dementia.
- Evaluation for differential diagnosis.
- Evaluation for early detection or pre-dementia, referred to as mild cognitive impairment (MCI), SPECT can detect a functional deficit and thus guide prognosis.
- Evaluation of patients for cerebrovascular disease.
- Preoperative localization of epileptic foci.
- Evaluation of traumatic brain injury (TBI), particularly if MRI and/or CT findings are unavailable. Blood flow abnormalities in TBI have been seen on SPECT even when anatomical scans appear normal, and SPECT findings are considered valuable for medical prognosis.
- Detecting and evaluating brain inflammation related to chronic inflammatory disorders such as HIV-encephalopathy, viral encephalitis, and vasculitis.
- Evaluating brain death.

Each of these indications—with the exception of brain death and the preoperative localization of epileptic foci—provide value to practicing psychiatrists who routinely evaluate and treat dementia, mental health symptoms related to brain trauma, and the consequences of cerebral vascular disease, infections, and inflammation.

In addition to the commonly accepted indications, the ESNM recommendations also report, “*SPECT can be useful in other indications such as movement disorders and psychiatric diseases (e.g., for follow-up of depression)*.”

## The Fundamentals of SPECT

**Figure 4:**
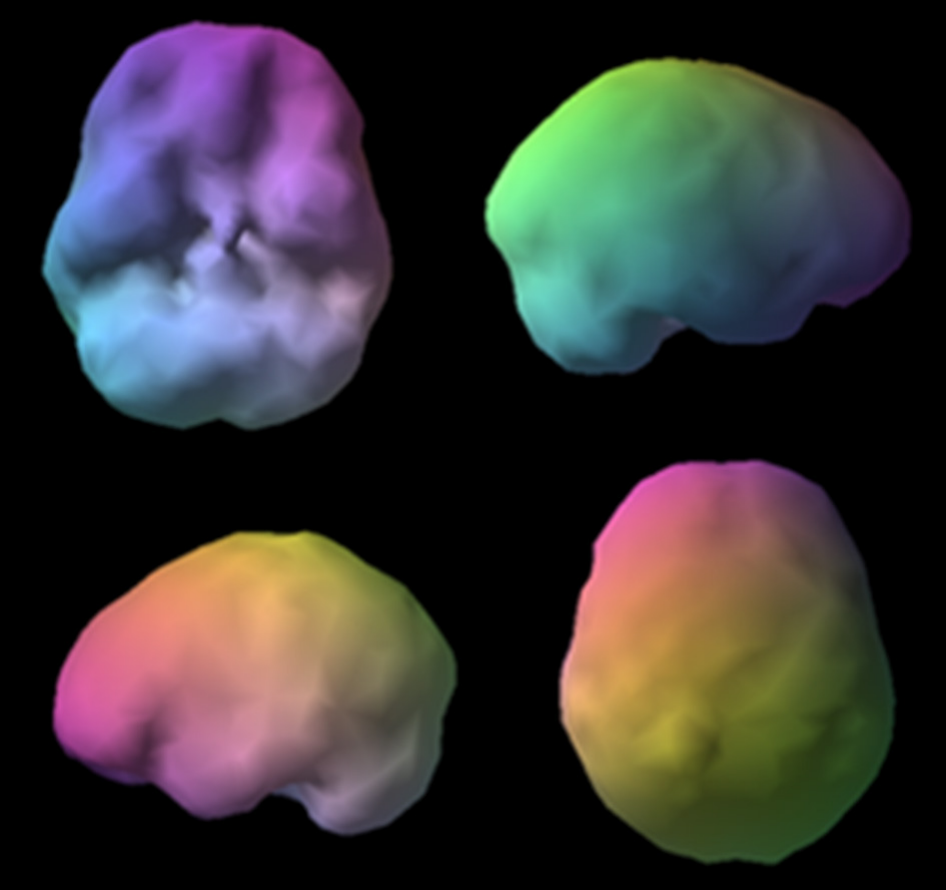
Normal surface rendering. A healthy 3D surface rendering of SPECT information, looking at the top 45% of brain perfusion. A healthy scan shows full, even, symmetrical perfusion.

**Figure 5:**
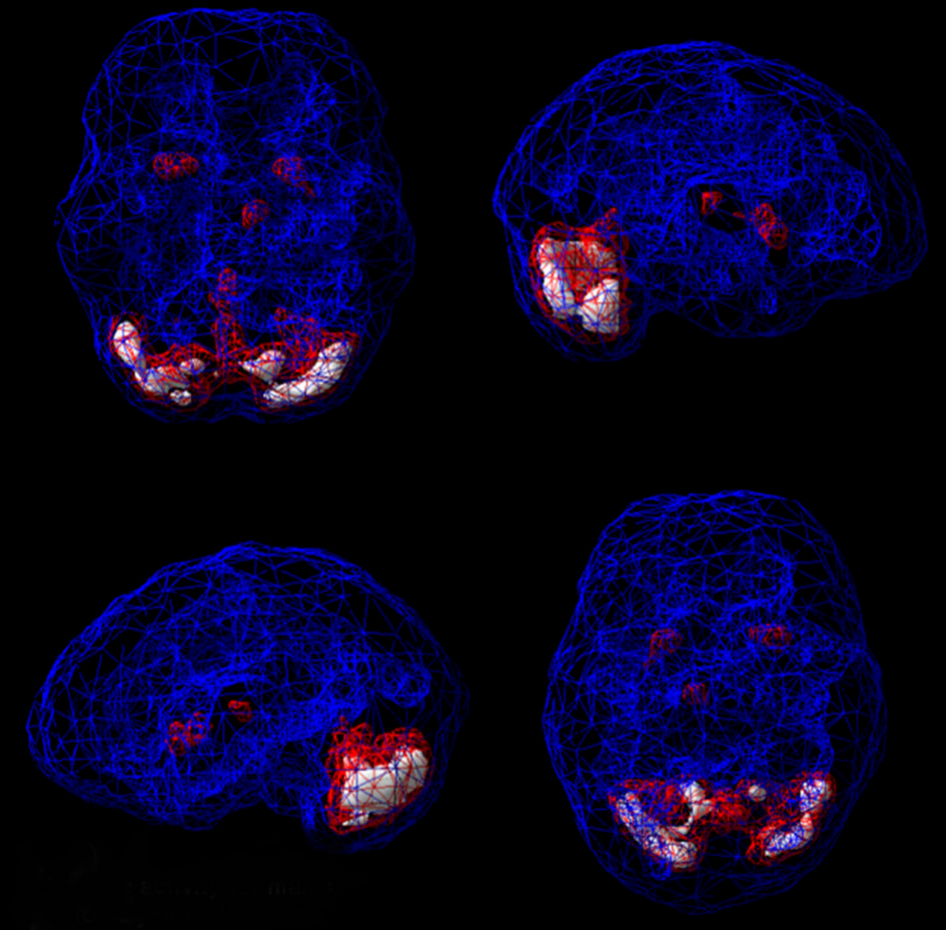
Normal active rendering. A healthy 3D active rendering of SPECT information, looking at areas of increased perfusion. Blue equals average perfusion, red equals the top 15% of perfusion, and white is the top 8%. A healthy active scan with HMPAO shows increased perfusion in the cerebellum.

SPECT is a nuclear imaging study that uses isotopes bound to ligands to measure rCBF and indirectly activity. Two common radiopharmaceuticals include HMPAO (Ceretec) and ECD (Neurolite); both provide images of rCBF, where each patient acts as their own control. The highest level of activity is typically seen in the cerebellum with HMPAO and the occipital lobes with ECD. Experienced clinicians look for symmetry and areas where perfusion is either increased and/or decreased. A SPECT scan of a healthy brain reveals full, even, symmetrical blood flow (30). See Figures 4, 5 for examples of healthy scans.

It is important to know a patient's age when evaluating their SPECT study, because rCBF changes as humans grow older. As a result of reduced myelinization and efficiency, children tend to have higher levels of activity in the brain compared to older adults who typically have decreased levels of perfusion.

In clinical practice, one problem that has been noted with SPECT is the variability of the imaging gamma cameras and resulting variability in image quality. Image quality can be significantly improved with multi-headed cameras and fan beam collimators. Multiple organizations, including the ACR, ESNM and the Society of Nuclear Medicine and Molecular Imaging (31), have each published similar procedure guidelines for SPECT.

## SPECT Has an Image Problem

One of the most commonly believed misconceptions about the clinical use of SPECT is that limited resolution restricts its value. In the early years of SPECT practice, single-headed cameras were utilized, producing low-resolution images, particularly in deep regions of the brain. For the past two decades however, sophisticated multi-headed gamma detectors with fan beam collimators have been available. According to George, multi-head SPECT camera resolution is comparable to PET at a much lower expense (32).

**Figure 6:**
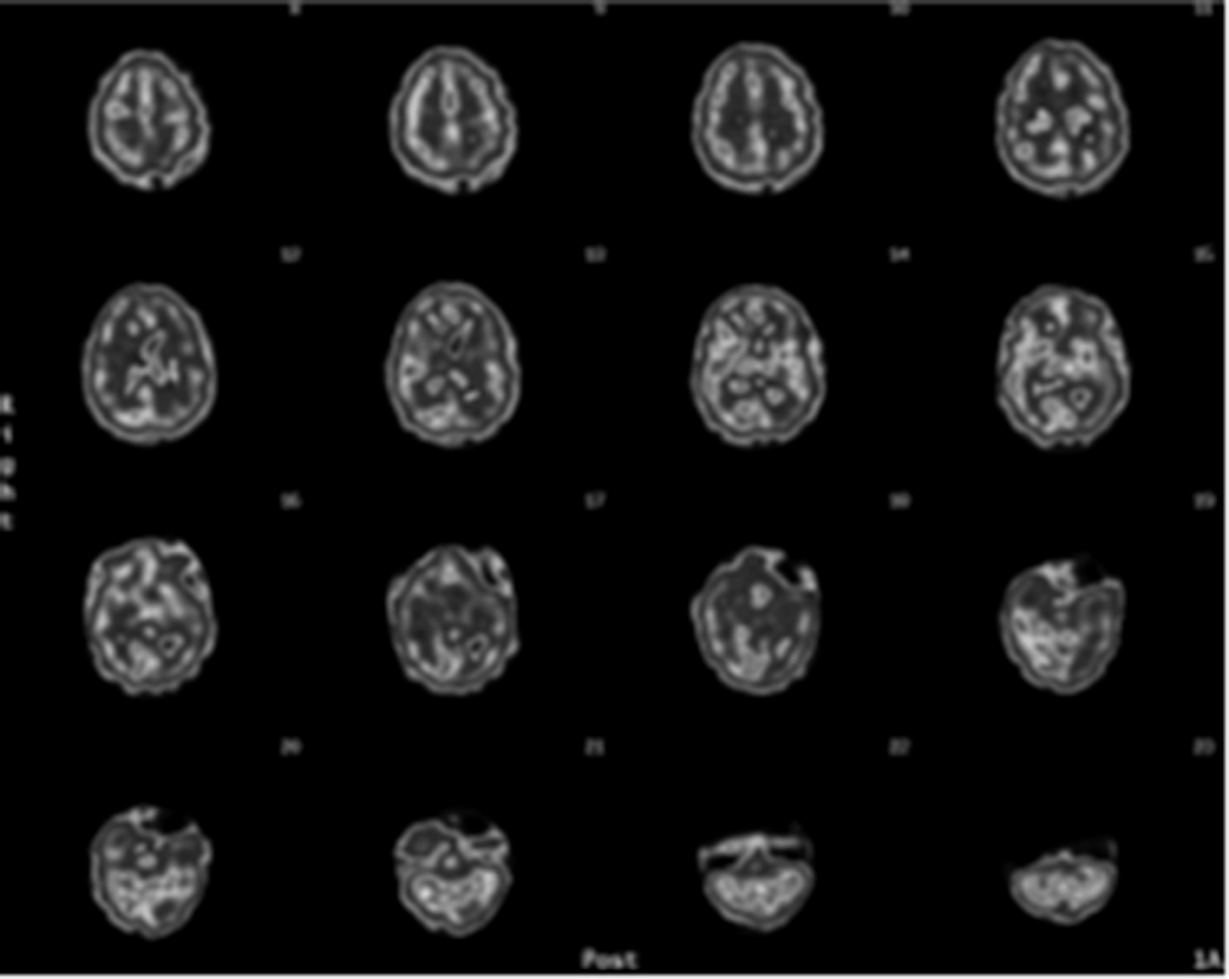
Typical gray scale SPECT rendered transaxial sliced images.

Another issue lies in the type of images provided by most nuclear medicine departments, namely they generate minute gray-scale coronal, sagittal, and horizontal SPECT slices (see Figure 6). These minuscule images are challenging to evaluate, including for nuclear clinicians who have years of experience. If radiologists or nuclear medicine departments provide images that clinicians find too difficult to interpret, those clinicians will consider the technology to be unhelpful. These challenges can be overcome with more advanced imaging options. For example, several manufacturers now provide software that produces three-dimensional image renderings—such as the images used in this article—that significantly enhance the images and make them far easier to interpret.

Another area of criticism surrounding SPECT lies in the potential for radiation exposure, particularly in children. For one single brain SPECT scan, the average radiation exposure is ~0.68 rem, which is less than a head CT (0.90 rem), which is ordered tens of millions of times each year in the U.S., and similar to a bone scan (33). Head CTs and bone scans are commonly ordered for a number of medical conditions, such as head injuries or bone fractures, indicating that such exposure to radiation is considered acceptable in medical practice. The American Academy of Neurology guidelines for SPECT report it is a safe procedure (34). To put the level of radiation from SPECT into perspective, in the U.S, natural background radiation exposure is 0.293 rem on the coast, while in the mountains of Colorado it is 0.387 rem. A single SPECT study is about twice the radiation from that of the natural environment along with other incidental radiation exposure that comes from traveling by plane, as well as from computers, televisions, and other devices (35).

## Overview of Common Brain Spect Imaging Patterns Relevant to Clinical Use

Based on the extensive volume of scientific literature on brain SPECT imaging, several key patterns have been detected that can be applied to clinical practice. Following, are seven of the most important examples:

### Overall Decreased Perfusion, or “Scalloping”

A pattern of overall decreased perfusion that has a scalloped or wavy appearance on scans is associated with exposure to toxins, certain forms of illness, or insult to the brain. This pattern is often associated with substance abuse (36, 37); the use of certain prescription medications, such as benzodiazepines^3^ (38); exposure to environmental toxins, such as carbon monoxide poisoning (39); infectious diseases, such as Lyme disease (40) or meningitis (41); severe hypothyroidism (42); anemia (43); anoxic states (44); hepatic encephalopathy (45); and exposure to general anesthesia (46, 47). The scalloping pattern does not indicate the etiology, rather it acts as an alert for clinicians to investigate further. Dr. Harold Bursztjan, who co-founded Harvard's Psychiatry and Law program, has said that SPECT scans do not provide answers, but rather they prompt clinicians to ask more pointed questions (48).

**Figure 7:**
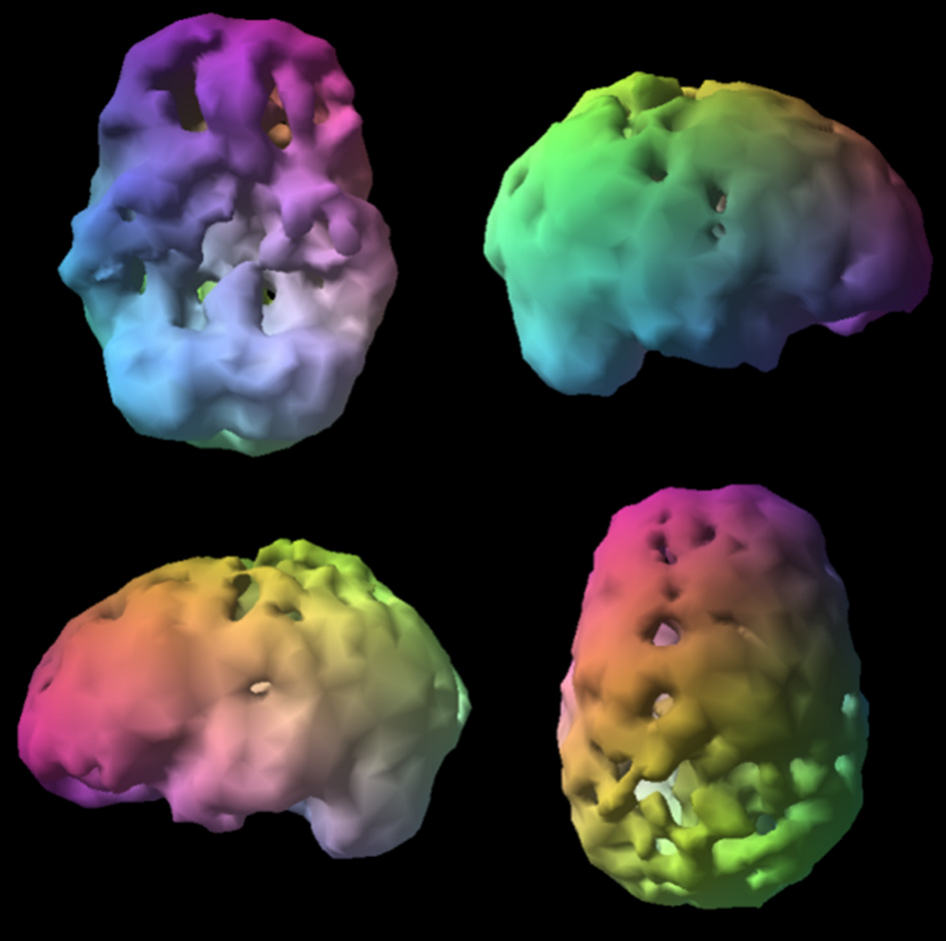
Toxic surface scan. Notice the scalloped, “Swiss cheese,” shriveled appearance indicating overall decreased perfusion. These are surface renderings of the SPECT information, looking at the top 45% of brain perfusion; anything below that level shows up as a hole or a dent. The holes do not mean no perfusion, they mean low perfusion, compared to a healthy dataset.

Consider this example:

A married couple came to Amen Clinics for evaluations after being told by their relationship counselor that they should end their marriage. This was after they had spent 3 years and thousands of dollars on therapy that wasn't effective. The problem appeared to be the husband who was diagnosed with mixed personality disorder including antisocial and narcissistic traits. The couple were upset by their therapist's suggestion to divorce and chose to seek a more thorough evaluation that included brain SPECT imaging. The husband's SPECT scan revealed overall reduced blood flow (Figure 7), which is often seen in substance abusers. However, the husband claimed that he didn't drink alcoholic beverages and had never engaged in the use of drugs. His wife confirmed that he didn't use alcohol or drugs. The scalloping pattern on his scan prompted his physician to question the man's personality disorder diagnosis. Upon further investigation, it was discovered that the husband was employed in a furniture factory, where he spent his days finishing cabinetry with toxic products. On scans, the use of inhalants is often associated with a toxic pattern (49). No amount of marital counseling would have helped this couple unless the husband's brain function improved. Removing him from the toxic environment at the furniture factory was an important step. The information about his brain dysfunction provided by the SPECT scans significantly altered his treatment plan and played a critical role in saving the couple's marriage.

### Imaging Patterns Seen in Traumatic Brain Injury (TBI)

TBI, a major cause of disability and death, has often been called a silent epidemic because of the devastating effects it can have on a person's life. Among the many issues TBI sufferers may face are a number of psychiatric disorders involving mood, functional status, and cognitive performance (50). It is clear that lingering symptoms do not occur in every person who has experienced a head injury. However, how could a psychiatric clinician know if a past brain injury may be contributing to mental health issues without looking at the brain using functional neuroimaging? A patient's clinical history doesn't tell the whole story. For example, many patients don't remember suffering a head injury. Other patients may recall past head trauma, but they are unaware that it can be linked to lasting mental health issues, so they don't mention it when discussing their clinical psychiatric history.

**Figure 8:**
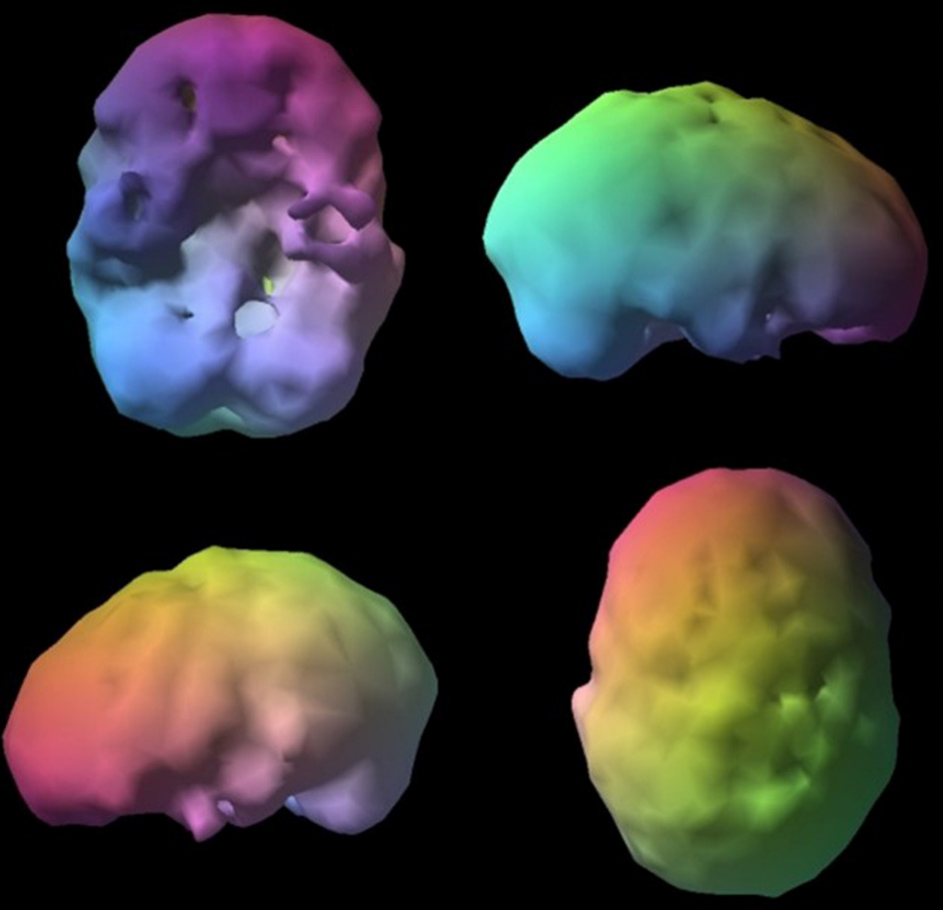
Brain trauma. Asymmetrical decreased perfusion in left frontal and temporal lobes.

As an example, one severely depressed and impulsive patient with addiction issues was asked 10 times if he has suffered a head injury. Each time, the 26-year-old male responded that he had not experienced any form of head trauma. In evaluating his SPECT scan (Figure 8), abnormal perfusion in the left frontal-temporal region indicated brain trauma. After seeing his own scan, the young man remembered being involved in an accident while riding a motorcycle that resulted in breaking his jaw on the left side of his face. The physical injury he sustained was near the site of the abnormal activity seen on his SPECT scan.

SPECT is helpful in the evaluation of brain trauma and in identifying the affected brain regions and systems. SPECT provides additional information that is useful to clinicians to improve their understanding of a patient's symptomatology as well as in recommending more targeted treatment plans (51). For example, on SPECT, abnormally low perfusion in the prefrontal cortex is often seen in patients with executive dysfunction, which may be improved with prescription psychostimulants or with additional strategies intended to increase PFC activity. As another example, seeing low levels of blood flow in the temporal lobe is commonly seen in patients struggling with mood instability and irritability and may see improvement with the use of anticonvulsant medication. Scientific research also points to SPECT's ability to measure abnormal perfusion levels in cases of mild head injuries, such as whiplash or post-concussion syndrome (52). Patients who have experienced head trauma but whose MRI, CT, and/or EEG scans are normal, frequently complain of headaches, forgetfulness and memory problems, difficulty concentrating, emotional lability, dizziness, and perceptual sensitivities. These patients are often called malingerers when, in fact, they are experiencing the lasting impacts of head trauma-related brain dysfunction that can be seen on SPECT. Researchers in the nuclear medicine field have analyzed the sensitivity of both structural and functional imaging tools in patients with mild to severe head injuries and have concluded that SPECT has greater sensitivity (53).

Comparing and contrasting the benefits and limitations of both structural imaging techniques and functional imaging tools as they relate to prognosis and clinical outcome of head trauma has been the subject of scientific research. Jacobs et al. used brain SPECT imaging in a prospective evaluation of 67 patients with mild, moderate, or acute brain injuries. All of the patients participated in an initial clinical evaluation and SPECT imaging within the first month of the head trauma and were re-evaluated and had a follow-up scan 3 months after their initial scan. On the initial SPECT scans, significant abnormalities were noted in 34 of the patients. Of these patients, 59% continued to report clinical symptoms 3 months later upon follow-up. Among the 33 patients whose initial SPECT scans showed no significant abnormalities, 97% reported during re-evaluation that their clinical symptoms had resolved within the 3-month period. The positive predictive value (PPV) of an initial SPECT scan with abnormalities was 59%, however if the repeat SPECT 3 months later also revealed abnormalities the sensitivity for the follow-up scan was 95%. According to these authors, negative initial SPECT scans can be “a reliable predictor of a favorable clinical outcome (54).”

Similarly, a 2014 systematic review of 52 cross-sectional and 19 longitudinal studies showed PPV increases from initial brain SPECT imaging performed soon after a head injury incident (PPV of 59%) to repeat scans after 12 months (PPV of 95%) (14). These results were replicated in subsequent studies involving a larger cohort. In cross-sectional and longitudinal studies, SPECT showed localization of lesions that were not detected on MRI or CT scans. In cross-sectional studies, the abnormalities seen on SPECT were most frequently seen in two brain regions: the frontal lobes (94%) and the temporal lobes (77%).

These findings show that SPECT can be useful in many areas of the clinical process in patients who have experienced varying degrees of head trauma. The functional brain imaging tool aids in making a diagnosis, determining a patient's prognosis, and developing an effective treatment plan in brain trauma survivors. In complex psychiatric cases, SPECT may also detect abnormalities associated with brain injuries even when the patient has no recollection of suffering a head trauma.

SPECT does have a limitation in its detection of brain trauma. In many cases, patients do not have a prior, or baseline, SPECT imaging study for comparison purposes. Thus, clinicians are unable to rely on neuroimaging to reveal the date when a trauma occurred. On functional imaging scans, brain trauma that occurred decades earlier or in childhood may look similar to trauma that happened more recently.

### Functional Neuroimaging Patterns Associated With Cognitive Decline

In the diagnosis of Alzheimer's disease (AD), autopsy reports that include an analysis of brain tissue remain the “gold standard” in the medical field. A growing body of scientific evidence, however, suggests that when evaluating patients who are presenting with cognitive decline, it may be helpful to use brain SPECT imaging in addition to diagnostic testing and clinical history (55). A number of common functional neuroimaging patterns have been noted in dementia patients: decreased perfusion in the parietal lobes, posterior cingulate gyrus, and medial temporal lobes are associated with AD; perfusion deficits in the frontal lobes and temporal lobes are seen in frontal lobe dementia; abnormalities in the occipital lobes are seen in Lewy body dementia (56); decreased vascular activity in several areas of the brain is associated with multi-infarct dementia; and decreased activity in the left prefrontal cortex combined with overactivity in the limbic system—a pattern typically associated with depression—is seen in pseudodementia (57). Clinicians are aware that patients with similar symptomatology (i.e., isolation and behavioral disinhibition) can have different dementia diagnoses. Considering that treatment protocols differ depending on the specific dementia disorder, differential diagnosis is vitally important. It is even more critical when patients presenting with dementia symptoms may actually be experiencing issues such as depression or normal pressure hydrocephalus that when treated appropriately resolve dementia-like symptoms. However, the wrong treatment can be devastating. For example, the use of antipsychotics for patients with hallucinations who actually have Lewy body dementia, can cause severe and sometimes irreversible deterioration.

**Figure 9:**
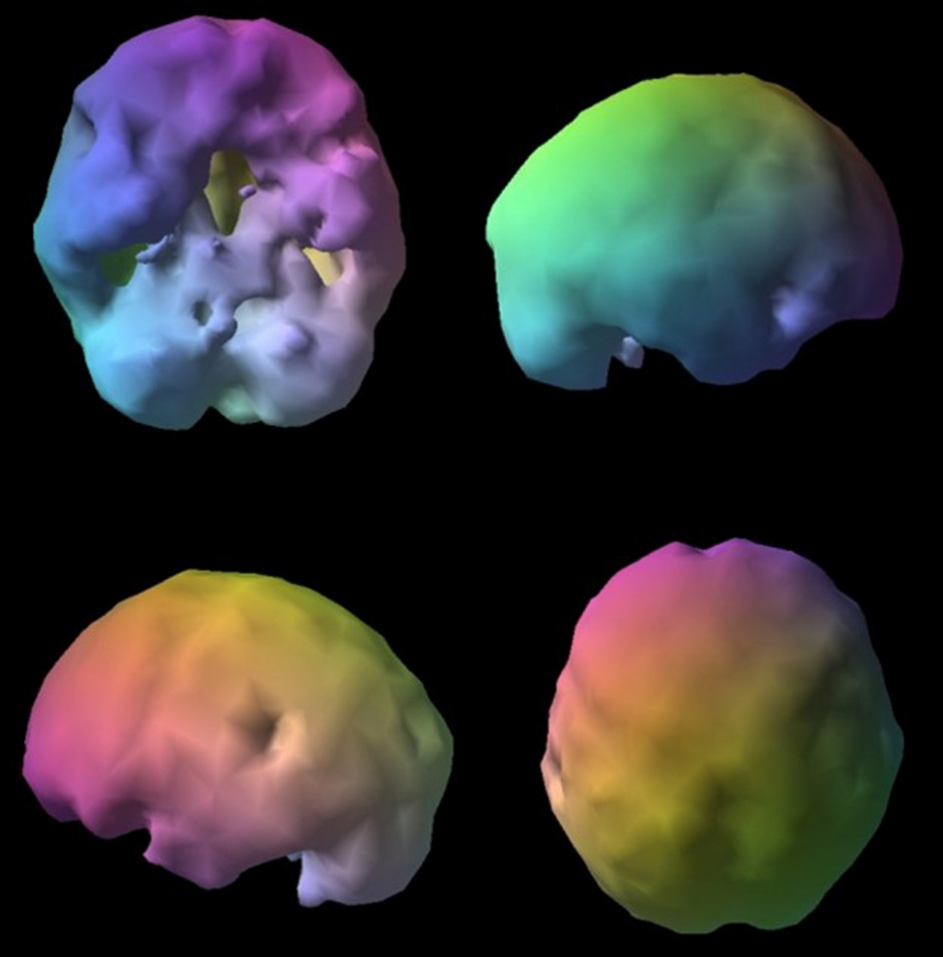
Surface SPECT images. Decreases in medial undersurface (see top left view).

**Figure 10:**
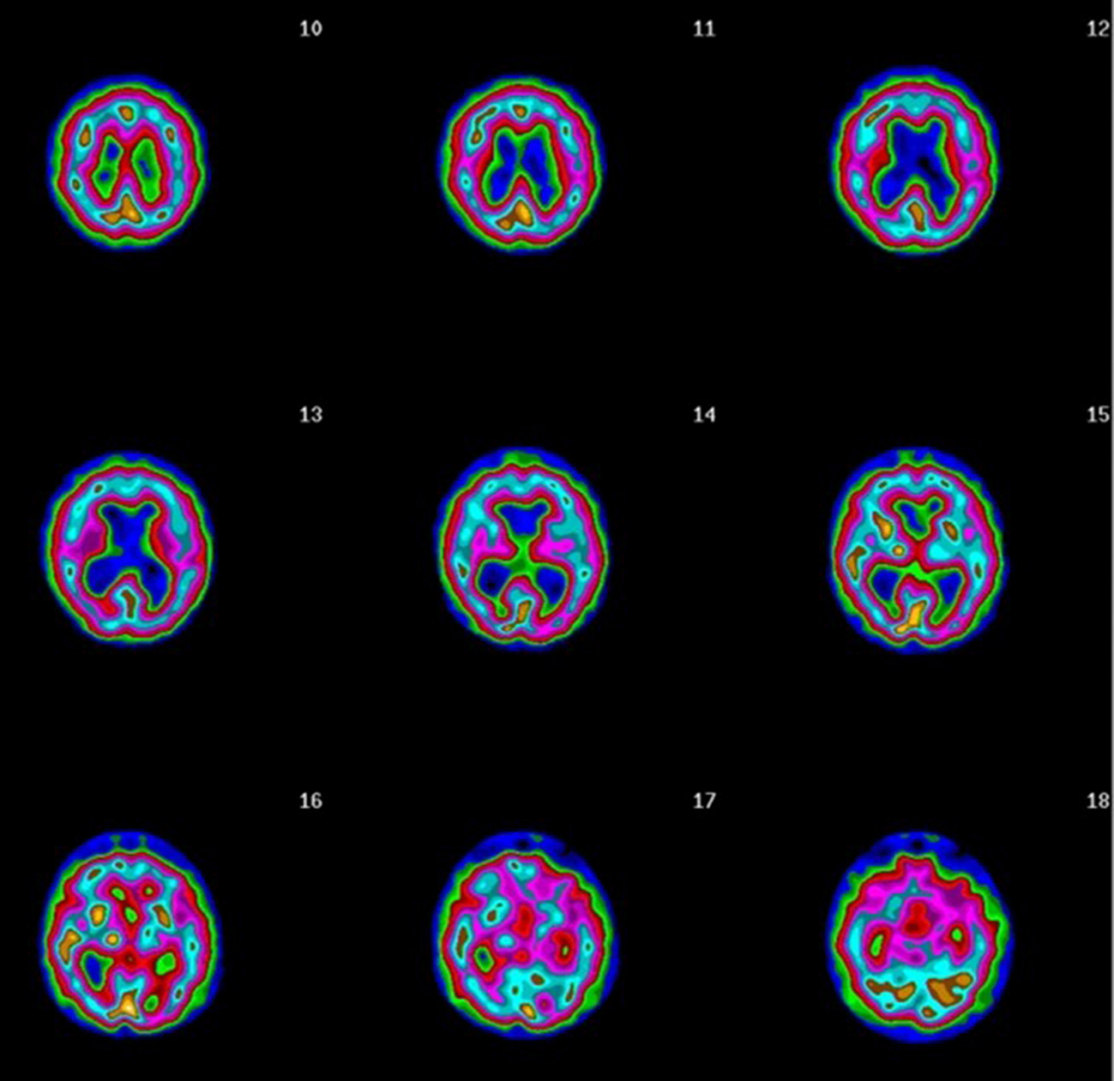
Transaxial slices. Slices 12 and 13 show inverted “lobster pattern” associated with ventricular enlargement.

For example:

L, a 73-year-old male with declining memory visited a neurologist for an evaluation that did not include functional brain imaging and was given a diagnosis of Alzheimer's disease. The commonly used medications, memantine and donepezil, however, did not improve his symptoms. Concerned about his deteriorating memory, his family brought him to Amen Clinics for a more complete evaluation including brain SPECT imaging. What the 73-year-old's SPECT scan revealed—significantly enlarged ventricles—was only a fraction of the story (see Figures 9, 10). What was *not* seen on the SPECT scan was even more telling. There was no hypoperfusion in the posterior cingulate and no bilateral decreased perfusion in the temporal and parietal lobes, which are commonly seen in AD. Based on the patterns seen on SPECT and on subsequent MRI neuroimaging, the man's diagnosis was changed from AD to normal pressure hydrocephalus. When treated appropriately for that diagnosis with the insertion of a shunt, he experienced marked improvements in his memory.

The results of SPECT studies in patients with suspected dementia to an elderly control group of healthy subjects were correlated with the histopathology of 54 subjects (biopsy in 3 and autopsy in 54) by Bonte et al. (58). The study revealed that the SPECT diagnoses were false-positive in 3 patients, false-negative in 6, true-positive in 37, and true-negative in 8 patients. Furthermore, the PPV was 92%, the sensitivity was 86%, and the specificity was 73%. These findings suggested that brain SPECT can be useful in both the early and late diagnosis of AD, as well as providing important differential-diagnosis information when the clinical presentation of demented patients is unclear or complicated.

Jobst et al. (59) evaluated functional (SPECT) and structural (CT) neuroimaging procedures for the utility of differential diagnosis in cases of dementia in comparison to clinical diagnoses of the cases. With 119 control subjects and 200 subjects with dementia, the study participants underwent annual medial temporal lobe CT scans and annual HMPAO SPECT studies The authors were able to conclude that the use of CT and SPECT together was more diagnostically accurate than standard clinical diagnosis. The study also found that common neuroimaging findings show parietotemporal hypoperfusion together with medial temporal lobe atrophy are common in AD but considerably less common in other types of dementia, and usually not found in normal controls.

DaTscan, another SPECT technology, has also been shown to help separate Alzheimer's disease from Lewy body dementia (60), and may have predictive value in longevity (61).

How does SPECT compare to PET in the diagnosis of dementia? Published studies of SPECT accuracy show that it is a useful tool for differential diagnosis, with sensitivities of 65–85% for diagnosing Alzheimer's disease and specificities (for other neurodegenerative dementias) of 72–87%. PET studies generally report slightly higher accuracies. However, there have been few direct head-to-head comparisons, with some indicating SPECT and PET to be equally useful in dementia. Although some studies suggest superiority of PET over SPECT, the evidence base for this is actually quite limited. Many of these studies have small numbers and methodically with poorly matched control groups (62). One must also take into consideration that SPECT scanning is more widely available and cheaper than PET. A new multi-headed SPECT gamma camera costs $300,000–800,000, while a PET scanner costs significantly more. Additionally, SPECT radio tracers are less expensive and have half-lives of up to 6 h, allowing a longer imaging time.

### Hyperfrontality

On SPECT, hyperperfusion or patterns of overactivity in the frontal lobes (anterior cingulate gyrus and prefrontal cortex) is called hyperfrontality. This functional neuroimaging finding is associated with a variety of psychiatric disorders involving cognitive inflexibility, which is seen in patients with a tendency to get stuck on negative or worrisome thoughts or unwanted or unhealthy behaviors. Hyperfrontality can cross several different diagnostic categories and can be seen in a variety of mental health conditions such as obsessive-compulsive disorder (OCD) (63), posttraumatic stress disorder (PTSD), autism spectrum disorder (ASD), some types of mood disorders, and certain types of anxiety disorders (64). Hyperfrontality is commonly noted in scans of patients who report being rigid, fixed, inflexible, argumentative, or oppositional and who have trouble adapting to new situations.

Brain imaging research shows that hyperfrontality on SPECT has been predictive of a positive treatment response to serotonergic medication in depression (65) and OCD (66), to sleep deprivation (67), and repetitive transcranial magnetic stimulation (68) for depression, and to a cingulotomy in OCD (69). Seeing hyperfrontality on SPECT scans also aids in differentiating ADHD from OCD (70).

It is important for clinicians to understand that hyperfrontality is not considered a diagnosis. However, seeing the biological underpinnings associated with the clinical symptomology helps provide necessary information that informs treatment.

**Figure 11:**
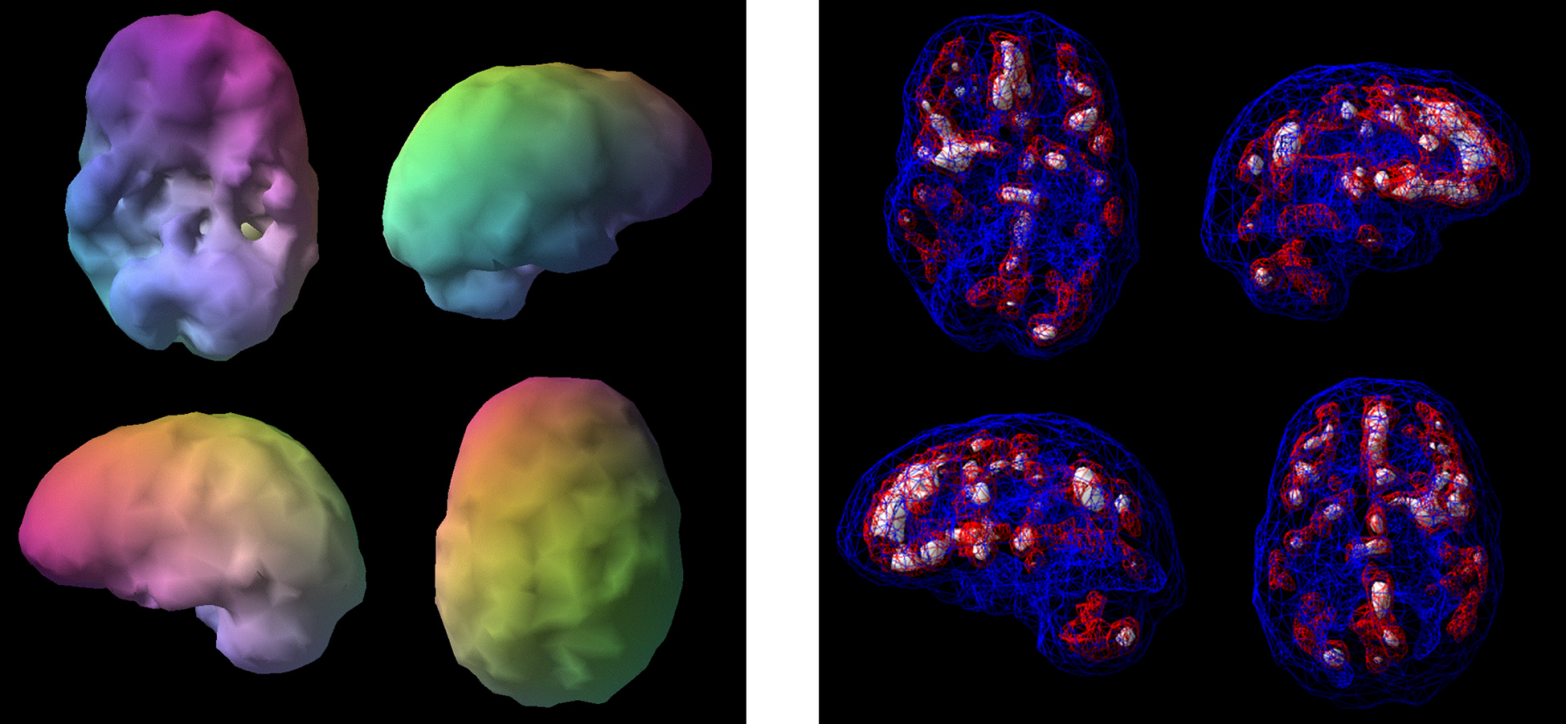
Surface views (L) and active views (R). Surface views (L) show severe temporal lobe hypoperfusion and active views (R) show severe hyperfrontality. The image on the left shows the outside surface of the brain, looking at the top 45% of brain activity. The image on the right shows the active rendering, where blue equals average perfusion, red is the top 15% of perfusion, and white is the top 8%. A healthy scan shows a symmetrical pattern with the most intense uptake in the cerebellum.

For example:

V, a 17-year-old male, presented with severe temporal lobe epilepsy, which developed following childhood meningitis. In addition, V displayed extreme aggression that had not responded to various behavioral interventions. His brain SPECT images showed severely reduced blood flow in his left temporal lobe (Figure 11 image on left), which is a common finding in epilepsy, and it also revealed extremely heightened perfusion in the anterior cingulate gyrus and lateral frontal lobes (Figure 11 image on right), which is often seen in obsessive-compulsive spectrum disorder. V's clinical history did not show symptomology typically associated with OCD; however, he did report being inflexible and rigid, and he had trouble coping when things did not go as planned. Amending his treatment plan by adding an antidepressant medication (escitalopram) that has been found to reduce overactivity in the frontal lobes significantly improved his behavior. Without the benefit of functional neuroimaging, it would have been more challenging to develop the most effective treatment protocol.

### Hypofrontality

Another common functional brain imaging pattern is hypofrontality, in which there is decreased blood flow or activity in the prefrontal cortex (PFC). This important finding can help clinicians better understand patient symptomology and more effectively target treatment to individuals. In scientific literature, hypofrontality is associated with improved response to stimulant medication in patients with ADHD (71) and improved response to acetylcholine-esterase inhibitors in Alzheimer's disease (72). In patients with depression, decreased perfusion in the PFC is predictive of a negative response to serotonergic antidepressants (73). In addition, decreased PFC perfusion is predictive of relapse in alcohol abusers (74) and is associated with impulsivity, antisocial behaviors, and homicide (75).

As research shows, hypofrontality is associated with a wide array of mental health issues, but it is not in and of itself a psychiatric diagnosis. Even so, it provides clinicians with critical information about abnormalities in brain function that can be beneficial in understanding and treating cognitive, emotional, or behavioral issues.

**Figure 12:**
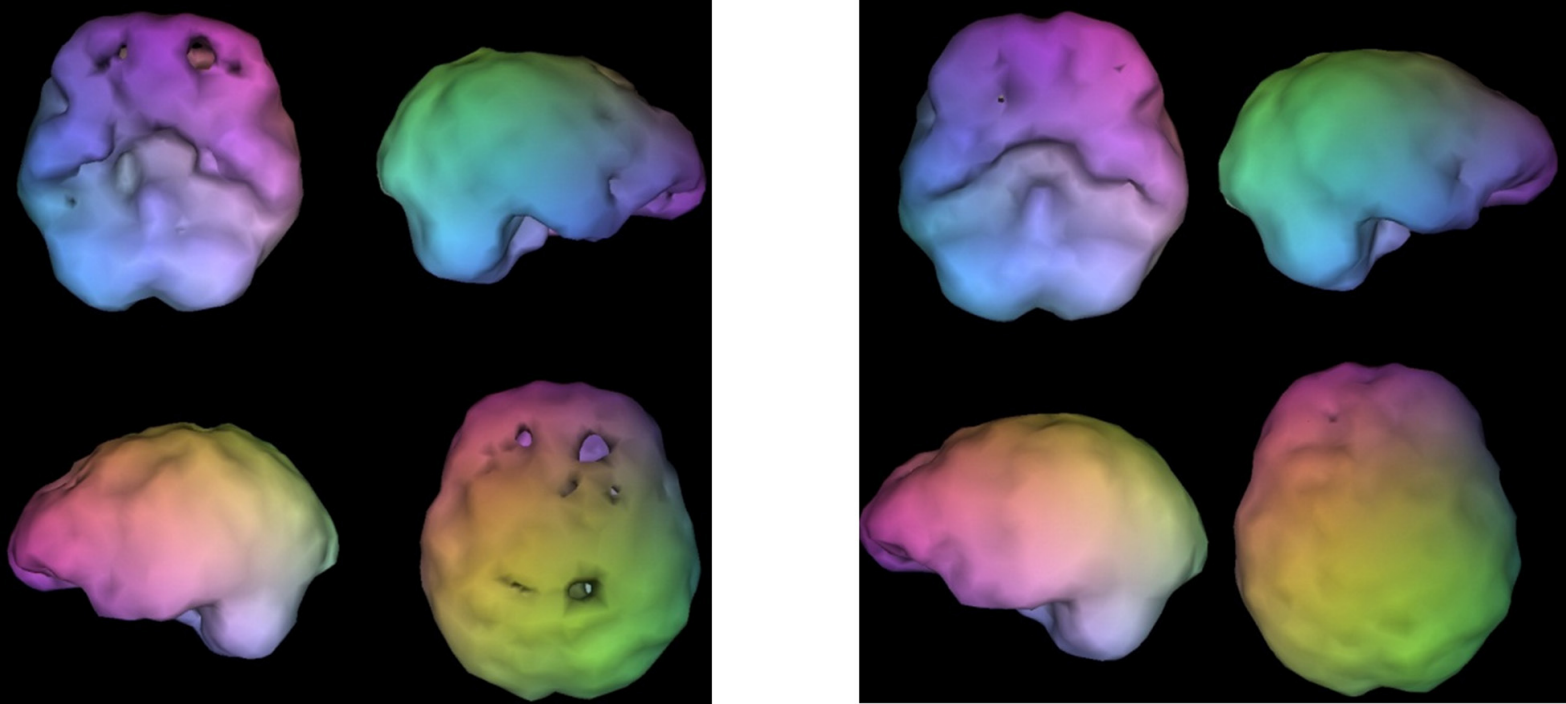
SPECT surface images without medication (L) and on mixed amphetamine salts (R). Without medication, SPECT images (L) reveal overall decreased prefrontal perfusion and post-treatment images (R) show improved prefrontal perfusion.

For example:

J, a 62-year-old female, had struggled with a lifetime of issues such as being inattentive, easily distracted, disorganized, and unable to maintain relationships. In her clinical history, she admitted that she had been fired multiple times for turning in work that wasn't always up to her potential and for being unreliable. Despite these troubles, she was resistant to seeking help even after a family member said she might have ADHD that could be treated. J said she was “too old to change.” With some prodding from her family, J eventually turned to Amen Clinics for an evaluation that included brain SPECT imaging. Her SPECT study showed significantly decreased PFC perfusion during a concentration task, which is a hallmark finding with ADHD. For her second SPECT scan, she took 10 mg of mixed amphetamine salts, which is a common ADHD treatment. The second scan showed a marked increase in perfusion to the PFC (see Figure 12). On treatment for ADHD, J experienced improvements in her cognitive function, as well as in her relationships and career. The neuroimaging scans not only aided the clinician but also helped the patient to understand that her symptoms had a biological basis that was treatable, which increased her compliance.

### Temporal Lobe Abnormalities

Abnormalities in temporal lobe perfusion are commonly seen in brain injuries and often relate to mood instability, temper problems, aggressive behavior, memory loss, and problems with language (76). This is why evaluating temporal lobe perfusion with a reliable neuroimaging tool such as SPECT is so critical in psychiatry. According to Devous et al. (77) “*Both SPECT and PET have localizing power approaching that of combined scalp and depth EEG.”* In psychiatry, it is important to localize potential seizure activity in patients who have epilepsy because they tend to have a high rate of co-existing mental health conditions (78). In addition, in the psychiatric field, anticonvulsants have become more commonly prescribed to stabilize moods. These medications have been found to improve overall perfusion and activity in the brain with particular improvement in the temporal lobes (79).

Temporal lobe epilepsy (TLE), a common chronic epileptic disorder, is associated with a broad range of psychiatric symptoms, such as depressed or euphoric mood, anxiety, fear, anergia, atypical pain, irritability, and insomnia (80). EEG is routinely used in the evaluation of epilepsy, however, this technology has difficulty evaluating certain key areas that are frequently involved in TLE, namely the medial aspects of the temporal lobes. By contrast, SPECT can show abnormalities that are undetected by EEG. The most common SPECT findings in epilepsy include focal reduced perfusion in the interictal phase and focal elevated perfusion in the ictal phase of a seizure. In a meta-analysis of 30 studies, Devous et al. found that sensitivities for SPECT localization in patients with temporal lobe seizures were 0.44 (interictal), 0.75 (postictal) and 0.97 (ictal). False-positive rates were low (81). These findings show that SPECT may be beneficial to clinicians in evaluating temporal lobe function in several ways, including the identification of abnormalities in any areas, finding deficits that go undetected by EEG, and potential insight into how and why anticonvulsants may be useful for a variety of neuropsychiatric indications.

Based on our three decades of brain SPECT imaging experience and clinical practice, we recommend anticonvulsants as a first-line treatment in patients who present with mood instability or anger and aggression problems and whose brain scans show either high or low perfusion in the temporal lobes. For patients with memory problems or difficulties learning whose brain scans reveal decreased perfusion in the temporal lobes, we consider acetylcholine-esterase inhibitors, provided it is deemed appropriate when the full clinical picture is considered.

Here is an example of how SPECT can be helpful in evaluating temporal lobe dysfunction:

J, a 17-year-old male, presented with severe mood swings, extreme temper problems, homicidal and suicidal thoughts, and a history of addiction. Despite evaluations by several psychiatric professionals, an 18-month stint in a residential treatment facility, and multiple medications, he wasn't improving. An addiction counselor suggested he might benefit from getting a SPECT scan.

**Figure 13:**
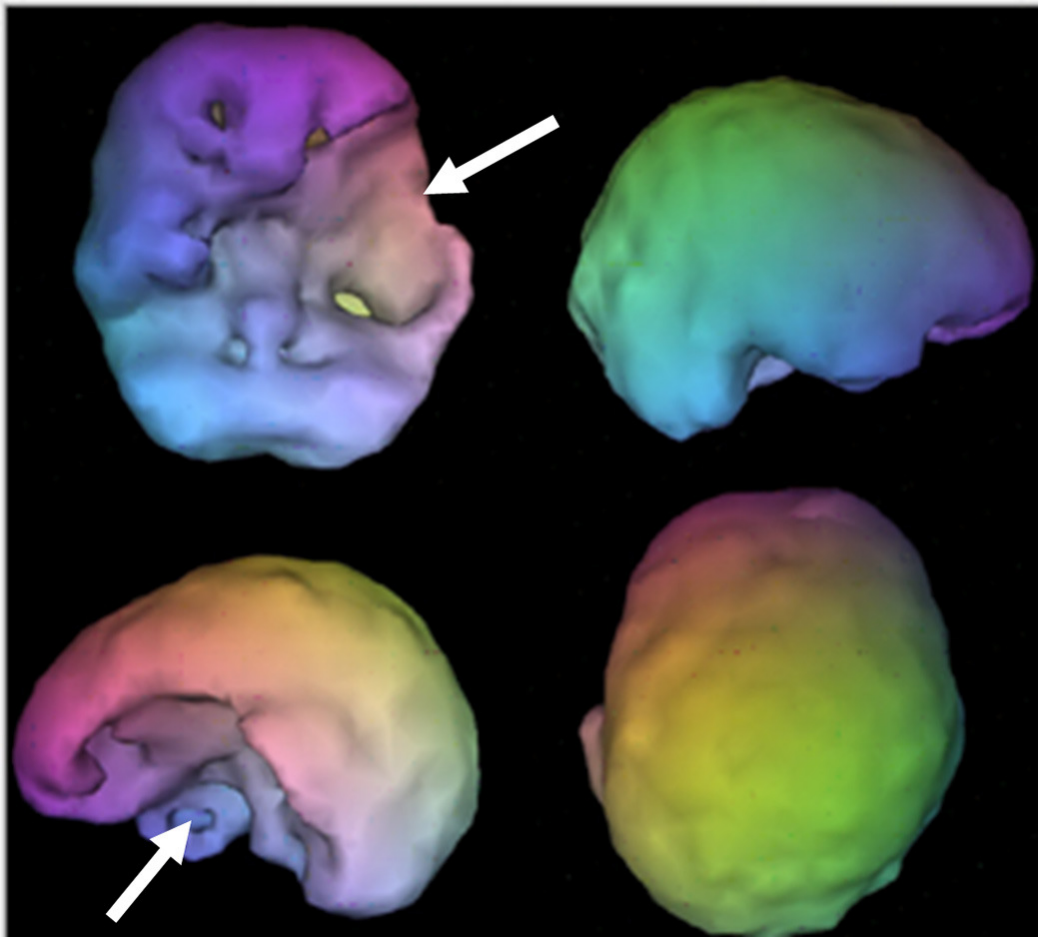
Tennis ball sized cyst occupying the left prefrontal and temporal regions. Images show large defect in left hemisphere.

His SPECT scan showed a major abnormality (Figure 13) in the form of a large defect cyst that took up nearly 25% of the left prefrontal and temporal regions of his brain. A subsequent MRI showed that inside his brain was a cyst the size of a tennis ball, which was causing compression of ventricles from a hemispheric shift. It is important to note that the results of J's neurological exam had been normal, but the SPECT scan clearly indicated that his brain was not normal.

Without the added information from the functional neuroimaging, most psychiatric professionals would give J multiple diagnoses, such as bipolar disorder and conduct disorder, which often leads to a subsequent diagnosis of antisocial personality disorder. J would then be placed on medications intended to treat those diagnoses and when those medications failed, psychiatrists might turn their attention to family therapy or other therapeutic strategies. But this is the wrong approach. It is clear that even the best family therapist wouldn't be able to help this teenager, nor would psychiatric medications. Unless the pressure in his brain was relieved, he would not get well.

Many psychiatric clinicians might argue that brain imaging with MRI would have sufficed in J's instance, and ultimately that proved to be the case. However, because the abnormality was clearly seen on SPECT, it is our opinion that SPECT is the preferred evaluation tool because it also provides valuable information on both increased and decreased perfusion, which MRI does not.

### Using Functional Neuroimaging to Subtype Dimensional Behavior

Using functional brain imaging in psychiatric clinical practice is in line with the efforts of the Research Domain Criteria (RDoC) initiative from NIMH, which aims to develop more effective ways to categorize psychopathology based on both observable behavior and objective neurobiological measures. Subtyping mental health conditions, such as ADHD, depression, and OCD based on brain dysfunction will be a critical component in finding the most effective and targeted treatment plans for individual patients.

Consider aggression, which the NIMH has identified as one of five key areas of interest in terms of brain function and is an area where SPECT can deliver immediate benefits to the clinician. Based on our brain imaging work on 75 murderers, as well as on hundreds of patients with a history of violent crimes such as bombings, rape, and kidnapping, we have seen that aggression is not associated with one single pattern in the brain, but rather clusters in three or more regions of the brain. One subtype is impulsive aggressive, which is associated with decreased perfusion in the frontal lobes (hypofrontality) and has been noted in the literature in antisocial personality disorder (82, 83). Patients with decreased perfusion in the PFC are often unable to control aggressive impulses, putting them at increased risk for violent behavior. Another subtype we have observed is compulsive aggressive, which is seen with increased perfusion in the frontal lobes (hyperfrontality). These patients have a tendency to explode because of inflexibility or negative thoughts get stuck in their mind. A third subtype of aggression we have seen in our brain imaging work is associated with abnormalities in the temporal lobes. In a retrospective analysis of SPECT and MRI scans from 21 individuals convicted of impulsive violent crimes, Soderstrom et al. (84) found that 16 of the offenders had varying levels of decreased perfusion in the temporal lobes and/or frontal lobes. The MRI scans of the violent criminals, however, did not reveal any anatomical problems or other abnormalities.

Since there is variability in the underlying cause of brain dysfunction in violent offenders, using SPECT to identify and understand any brain abnormalities can prompt the psychiatric clinician to ask additional questions that can lead to a more individualized and effective treatment protocol for managing symptoms and behavior problems.

## Summary

By identifying a patient's specific brain system dysfunction, SPECT can provide important diagnostic information that helps to individualize treatment protocols to each patient's unique needs. The standard practice of relying solely on traditional diagnostic procedures and therapeutic interventions without imaging data in complex cases has been found to be less accurate and with diminished remission rates. We have described 7 clinical areas in this paper that illustrate the complexity of symptoms relative to brain function. The authors' experiences have found that a secondary benefit of using brain SPECT imaging is that by seeing images of their brains, the patients and their families are less burdened by the stigma of mental illness and the guilt and shame that accompany it. As such, they can see that their psychiatric illnesses have medical underpinnings and are not moral shortcomings. This in turn has led to greater compliance with treatment. Plus, after seeing their own scans, patients develop a personal relationship with their own brains and often desire to treat it better, including the avoidance of toxins, like recreational drugs and alcohol. After seeing his scan, one patient said, “I felt the same after seeing one of my children for the first time; I knew I would never do anything again to hurt it.”

As with all neuroimaging modalities, the findings from brain SPECT studies are not a stand-alone diagnosis but should always be incorporated into the overall clinical assessment. There have been many discussions over the course of the last 30 years with regard to the “Future of Psychiatry” (85) as medicine begins to adopt a more biologically-based paradigm. Within this new framework, some are concerned about being able to maintain a subjective focus on patients. Reynolds, Lewis, et al. (86) opined that it is vital to ensure resident trainees have ongoing exposure to translational neuroscience early on in their residencies, and that neuroimaging is included in the curriculum. A recent report (87) demonstrated the integration of a neuroimaging module within a residency program had wide approval by residents. Certain programs have included neuroimaging to some extent during the preliminary year of the internal medicine neurology rotation. The further development of this field will need to be taken on by the Resident Review Committee for the psychiatry branch of the Accreditation Council for Graduate Medical Education (88).

The field of psychiatry is likely to continue evolving over the course of the next three decades as it increasingly incorporates functional neuroimaging in clinical practice. Quantitative EEG, arterial spin labeling, PET, and other modalities are bolstering the arsenal of neuroimaging for the future.

In the early 1990s, the APA spent several years providing courses on SPECT, but ultimately dismissed the use of neuroimaging, because it threatened the perceived integrity of the DSM which they own and from which they make money (6). Sadly, the limitations of the APA's position completely overlooked the benefits that neuroimaging tools have for physicians and their patients. Because psychiatry is the only medical specialty that does not use objective visual data for the organ they treat, they are relegated to diagnosing complex patients by looking for symptom clusters deduced from talking to and looking at them. In 1840, that was the methodology Dr. Anson Henry used to diagnose Abraham Lincoln with melancholia.

The original DSM was created prior to the development of functional assessment tools, such as SPECT and qEEG, thus the DSM was not—and is not—based on underlying neuroscience. Because of this, physicians who include functional brain imaging in clinical practice understand that scans will never match up with the siloed categories of the DSM. Experienced clinicians can often tell, in simple cases, if some individuals are likely to have ADHD, OCD, or bipolar disorder without the benefit of these tools. Given the high overlap of symptoms in more difficult cases the appropriate diagnosis is frequently missed by looking only at symptom clusters. When misdiagnosed, the treatment implications in these clinical cases can be devastating. It has been our experience that looking at brain systems using functional neuroimaging such as SPECT is extremely helpful in aiding us in these more complicated presentations.

*Without the use of functional brain imaging, it is impossible to identify any brain pathophysiology of the patients they treat*. Without imaging the brain, psychiatrists would not even consider if a patient's inattention, depression, or aggression could be from:

➢ Toxic exposure
➢ Head trauma
➢ Early dementia process
➢ Hyperfrontality
➢ Hypofrontality
➢ Temporal lobe abnormalities
➢ Other potential causes

If we don't look at the brain, we are unnecessarily flying blind ***and will*** miss important diagnoses, and be led to the wrong treatment plan, potentially resulting in years of patients' suffering, disability, and potential suicide. This is a disservice to our patients and these errors can be minimized so we don't hurt the people and families we are entrusted to help.

The authors believe clinicians should consider ordering SPECT scans in complex cases, where traditional approaches prove to be ineffective. This will help clinicians move away from treating symptoms to looking at and treating dysregulation of various brain systems. Additionally it will help psychiatrists expand their recognition, understanding, and treatment of other comorbid factors affecting treatment efficacy such as TBI, infection, inflammation, and toxicities. In a large outcome study we performed at Amen Clinics on over 500 patients, before coming to us they failed 3.3 providers and 6 psychiatric medications (89). We suggest a SPECT scan should be considered after two treatment failures.

Be careful if you wish to change a paradigm, as it invites cruel and bitter criticism^4^, which has been our experience. In the fifteenth century, the Italian politician, Niccolo Machiavelli, explained: “*It must be remembered there is nothing more difficult to plan, more doubtful of success, nor more dangerous to manage than a new system. For the initiator has the enmity of all who would profit by the preservation of the old institutions…”* (90).

## Data Availability Statement

The original contributions presented in the study are included in the article/supplementary material, further inquiries can be directed to the corresponding author/s.

## Ethics Statement

For all case studies presented herein, written informed consent was obtained from the individual adult patients as well as from the minors' legal guardians for the publication of any potentially identifiable images or data included in this article.

## Author Contributions

All authors listed have made a substantial, direct, and intellectual contribution to the work and approved it for publication.

## Conflict of Interest

DA is founder and owner of Amen Clinics, Inc. The remaining author declares that the research was conducted in the absence of any commercial or financial relationships that could be construed as a potential conflict of interest.

## Publisher's Note

All claims expressed in this article are solely those of the authors and do not necessarily represent those of their affiliated organizations, or those of the publisher, the editors and the reviewers. Any product that may be evaluated in this article, or claim that may be made by its manufacturer, is not guaranteed or endorsed by the publisher.

## Footnotes

^1^ https://en.wikiquote.org/wiki/Max_Planck

^2^ http://www.amenclinics.com/brain-science/spect-research/spect-abstracts/

^3^ https://www.psychiatrictimes.com/view/anti-youth-pill-alprazolam-toxicity-can-add-years-brain-age-and-appearance

^4^ https://www.washingtonpost.com/lifestyle/magazine/daniel-amen-is-the-most-popular-psychiatrist-in-america-to-most-researchers-and-scientists-thats-a-very-bad-thing/2012/08/07/467ed52c-c540-11e1-8c16-5080b717c13e_story.html
